# DEAD-ly Affairs: The Roles of DEAD-Box Proteins on HIV-1 Viral RNA Metabolism

**DOI:** 10.3389/fcell.2022.917599

**Published:** 2022-06-13

**Authors:** Shringar Rao, Tokameh Mahmoudi

**Affiliations:** ^1^ Department of Biochemistry, Erasmus University Medical Centre, Rotterdam, Netherlands; ^2^ Department of Pathology, Erasmus University Medical Centre, Rotterdam, Netherlands; ^3^ Department of Urology, Erasmus University Medical Centre, Rotterdam, Netherlands

**Keywords:** DEAD-box proteins, HIV-1, viral RNA metabolism, DEAD-box protein inhibitors, novel antiretrovirals

## Abstract

In order to ensure viral gene expression, Human Immunodeficiency virus type-1 (HIV-1) recruits numerous host proteins that promote optimal RNA metabolism of the HIV-1 viral RNAs (vRNAs), such as the proteins of the DEAD-box family. The DEAD-box family of RNA helicases regulates multiple steps of RNA metabolism and processing, including transcription, splicing, nucleocytoplasmic export, trafficking, translation and turnover, mediated by their ATP-dependent RNA unwinding ability. In this review, we provide an overview of the functions and role of all DEAD-box family protein members thus far described to influence various aspects of HIV-1 vRNA metabolism. We describe the molecular mechanisms by which HIV-1 hijacks these host proteins to promote its gene expression and we discuss the implications of these interactions during viral infection, their possible roles in the maintenance of viral latency and in inducing cell death. We also speculate on the emerging potential of pharmacological inhibitors of DEAD-box proteins as novel therapeutics to control the HIV-1 pandemic.

## 1 Introduction

37 million people worldwide are currently infected with Human Immunodeficiency Virus type-1 (HIV-1), the causative agent of acquired immune deficiency syndrome (AIDS). Although combination antiretroviral therapy (cART) can suppress viral replication, this treatment is not curative and cessation of therapy results in the rebound of viremia (Finzi et al., 1997). People living with HIV-1 (PLWHIV) therefore need to receive lifelong cART to control HIV-1 replication, while the emergence of drug resistance (Günthard et al., 2019) compromises the efficacy of cART. Currently, one in four PLWHIV has no access to antiviral treatment, highlighting the need for HIV-1 curative therapies and the development of newer classes of antiretrovirals with long-lasting activity. HIV-1 is an obligate intracellular parasite that utilises host proteins for its replication. Therefore, delineating the underlying molecular mechanisms of the interactions between cellular host factors and HIV-1 will be critical not only to understanding how different steps of the HIV-1 life cycle are regulated but may unravel novel molecular targets or pathways for pharmacological inhibition.

HIV-1 is a lentivirus whose life cycle begins after binding of the viral particle to receptors on the surface of CD4^+^ cells, followed by fusion of the viral envelope with the host cell membrane, allowing the release of the two copies of HIV-1 genomic viral RNA in the cell (Chen, 2019). The HIV-1 RNA genome is then reverse transcribed into double-stranded DNA using the viral enzyme reverse transcriptase (RT) (Hu and Hughes, 2012). The double-stranded HIV-1 DNA constitutes the main component of the HIV-1 pre-integration complex which then enters the nucleus where it integrates into the host genome aided by the activity of the viral enzyme Integrase (IN). The integrated HIV-1 double stranded DNA then behaves essentially as a cellular gene, using the cellular transcription, RNA-processing and translation machinery to initiate promoter transcription, mRNA splicing, processing and export of the viral RNAs, as well as translation into viral proteins, which are cleaved by the viral protease enzyme into maturity (ref). (Mailler et al., 2016).

During active replication, transcribed, viral RNAs (vRNAs), similar to host cell mRNAs, need to undergo multiple processes involved in RNA metabolism to ensure viral gene expression. Alternative splicing of HIV-1 results in the generation of three species of RNAs: unspliced viral RNA (US vRNA), singly-spliced HIV-1 viral RNA (SS vRNA) and multiply spliced viral RNA (MS vRNA). US vRNA serves as the genomic RNA and is packaged into the budding viral particles during viral assembly. US vRNA codes for the main HIV-1 structural and enzymatic polyproteins, Gag and Gag-Pol, which are further cleaved by the viral-encoded protease to yield viral proteins, including the p24 capsid protein. SS vRNAs code for the viral accessory proteins Vif, Vpr, Vpu, and for the envelope polyprotein Env. MS vRNA codes for the viral proteins Tat, Rev and Nef (Fields, 2013). Tat plays a very important role in the transcription of the proviral DNA by recruiting host positive transcription elongation factor b (P-TEFb) to the HIV-1 promoter (Ali et al., 2021). The viral protein Rev is critical for the nucleocytoplasmic export of the US and SS vRNAs. Rev binds to the US and SS vRNAs on a region known as the Rev-response element (RRE) thereby regulating their nucleocytoplasmic export via the Chromosomal Maintenance 1 (CRM1) pathway (Taniguchi et al., 2014). Nef is a viral protein that regulates viral infectivity of progeny virion and disease progression (Basmaciogullari and Pizzato, 2014). All the vRNAs need to be trafficked to the right subcellular localisation and be present in specific ribonucleoprotein complexes (RNPs) conducive to their translation to ensure viral replication. These various processes of RNA metabolism are mediated by many families of host RNA-binding proteins, including the DEAD-box helicases.

While combination antiretroviral therapy (cART) effectively abrogates active viral replication, viral latency occurs in a minority of infected cells, which constitute the HIV-1 reservoir. The development of viral latency is the main barrier to the development of an HIV-1 cure. Latency results from a block in viral gene expression, despite the presence of an intact replication-competent integrated provirus. While this block has been extensively investigated at the level of chromatin-mediated repression, transcription initiation and transcription elongation, more recently, it has come to light that significant blocks also exist at the level of RNA splicing and processing resulting in a block in the translation of viral proteins (Yukl et al., 2018). DEAD-box proteins could influence the maintenance of viral latency by influencing these post-transcriptional stages of HIV-1 replication, making them possible targets in the development of curative HIV-1 therapies.

DEAD-box helicases are a family of proteins that play pivotal roles in RNA metabolism, including transcription, splicing, trafficking, ribosome biogenesis, translation and turnover (reviewed in (Rocak and Linder, 2004; Linder and Jankowsky, 2011)). HIV-1 does not encode its own helicase and must therefore rely on host cell proteins to ensure its gene expression. Over the last 2 decades, it has come to light that the DEAD-box proteins play multiple roles in the HIV-1 life cycle. In this review, we discuss all DEAD-box proteins that have been implicated to play a role in HIV-1 replication.

## 2 DEAD-Box Proteins

**FIGURE 1:**
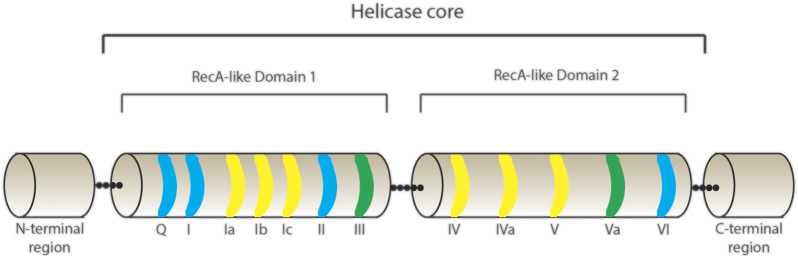
Schematic representation of the helicase core of DEAD-box proteins. The helicase core is composed of two RecA-like Domains 1 and 2 connected by a linker region. The conserved motifs presented are highlighted in blue for domains with ATP-binding and hydrolysis activity, yellow for RNA binding activity and green for domains involved in communication between ATP and RNA - binding sites.

The proteins belonging to the DEAD-box family are adenosine triphosphate (ATP)-dependent RNA helicases that use energy from ATP hydrolysis to remodel RNA or RNPs, and thus facilitate multiple steps of RNA metabolism. They derive their family name from the presence of a DEAD-box amino acids sequence of Asp (D)–Glu (E)–Ala (A)–Asp (D) in all proteins in this family (Linder et al., 1989). The DEAD-box family belongs to the superfamily 2 (SF2) of nucleic acid helicases and contains the most number of proteins for a family of helicases, with more than 35 DEAD-box proteins present in humans. DEAD-box proteins share a highly conserved core with at least twelve conserved motifs that contain regions involved in RNA-binding, ATP binding and hydrolysis (Figure 1) (Caruthers and McKay, 2002). Two RecA-like domains make up the helicase core that is connected by an adaptable linker that facilitates their transition from open and closed conformations, the latter being associated with enzymatic activity (Ali, 2021). The binding of the DEAD-box proteins to RNA is sequence-independent (Andersen et al., 2006), important for mediating the binding of DEAD-box proteins to multiple RNAs and modulating numerous cellular functions. Although they are broadly categorised as helicases, DEAD-box proteins do a lot more than just unwind RNA; they are involved in almost every step of RNA metabolism (Linder and Jankowsky, 2011). They play fundamental roles in almost every step of gene expression (reviewed in (Bourgeois et al., 2016). Table 1 depicts a summary of the DEAD-box proteins involved in different steps of gene expression and indicates which of these proteins have also been implicated in HIV-1 gene expression. More than 30 members of the DEAD-box family of proteins have been reported to play a role in HIV-1 replication. This suggests that, just like for host genes, the DEAD-box family of proteins is also involved in almost every stage of viral RNA gene expression.

**TABLE 1:** List of DEAD-box helicases involved in different steps of gene expression. The proteins with a well-characterised role in HIV-1 replication are shaded in green and those that have been implicated in playing a role in viral replication are shaded in yellow.

Transcription	Splicing	Nucleo-Cytoplasmic Export	Translation Inhibition and RNA Decay	Cytoplasmic Trafficking and Storage	Translation Promotion
DDX5	DDX1	DDX1	DDX1	DDX1	DDX2A
DDX17	DDX3	DDX3	DDX4	DDX3	DDX2B
DDX20	DDX5	DDX5	DDX5	DDX4	DDX3
DDX21	DDX17	DDX6	DDX6	DDX5	DDX4
—	DDX23	DDX19	DDX20	DDX6	DDX6
—	DDX39B	DDX21	DDX48	DDX19	DDX19
—	DDX41	DDX25	—	DDX20	DDX25
—	DDX42	DDX39A	—	DDX25	DDX43
—	DDX46	DDX39B	—	DDX39B	—
—	DDX48	DDX48	—	DDX41	—
—	—	DDX49	—	—	—
—	—	DDX56	—	—	—

## 3 Roles of DEAD-Box Proteins in HIV-1 Replication

Over the last years, multiple transcriptomic, proteomic and cell-based assays demonstrate that DEAD-box proteins play even more pivotal roles during HIV-1 infection, such as in their direct recruitment by the virus to facilitate replication, or in the modulation of innate immune responses to HIV-1. Helicases belonging to other families such as RHA (Roy et al., 2006), UPF1 (Ajamian et al., 2008) and MOV10 (Burdick et al., 2010) are all known to influence HIV-1 replication. In the following section, we focus specifically on the members of the DEAD-box family and discuss their emerging roles in the regulation of various facets of the HIV-1 life cycle and gene regulation.

### 3.1 DEAD-Box Helicases With Well-Characterised Roles in HIV-1 Replication

Ever since the first evidence in 2004 when DDX3 was identified as a crucial co-factor for Rev-mediated vRNA export, DEAD-box proteins and other RNA helicases have been studied in the context of the HIV-1 life cycle (reviewed in (Ajamian et al., 2008; Lorgeoux et al., 2012; Chen et al., 2013)). In the following sub-section, we will discuss the most-investigated DEAD-box helicases in the context of the HIV-1 life cycle with characterised functions in viral replication.

#### 3.1.1 DDX3

Historically, DDX3 is the first DEAD-box protein family member to have been implicated to be involved in HIV-1 RNA regulation. DDX3 X-Linked (DDX3X), herein referred to as DDX3, is a host protein involved in all aspects of RNA metabolism including RNP assembly, pre-mRNA splicing, nucleocytoplasmic transport and translation, as well as in cell cycle progression, stress response, innate immune sensing and apoptosis (Linder and Jankowsky, 2011; Soto-Rifo and Ohlmann, 2013; Zhao et al., 2016). It contains a nuclear export signal (NES) that facilitates its shuttling between the nucleus and the cytoplasm. Because of DDX3’s characterised roles in several cancers, playing a dual role in cancer progression because of both its oncogenic as well as tumour suppressive activities, DDX3 has emerged as a promising target in cancer therapy (Bol et al., 2015b; Heerma van Voss et al., 2017; He et al., 2018; Lin, 2019; Kukhanova et al., 2020). A less characterised paralog, DDX3Y is located in the non-recombining region of the Y chromosome. In the context of HIV-1 infection, DDX3 plays multiple critical roles to ensure viral replication (reviewed in (Hernández-Díaz et al., 2021)). DDX3 plays an integral role in the nucleocytoplasmic export of the US vRNA by facilitating the CRM1-mediated export of the intron-containing unspliced and singly spliced vRNA-transcripts (Yedavalli et al., 2004; Mahboobi et al., 2015). Depletion of DDX3 resulted in impaired Rev-RRE function and reduction in viral production (Yedavalli et al., 2004). Additionally, DDX3 is involved in the initiation of translation of HIV-1 genomic mRNA by promoting the unwinding of RNA structures close to the 5′Cap structure to facilitate the loading of the 43S preinitiation complex on the US vRNA (Soto-Rifo et al., 2012; Soto-Rifo et al., 2013; Frohlich et al., 2016). Using GST-pull down studies, DDX3 has been shown to interact with the HIV-1 protein Tat to enhance the translation of 5ÚTR-containing RNAs (Lai et al., 2013). DDX3 has also been implicated in the function of Tat in the activation of HIV-1 transcription (Yasuda-Inoue et al., 2013a). In the context of viral latency, a depletion or pharmacological inhibition of DDX3 resulted in viral reactivation in T-cell derived cell lines, primary CD4^+^ T cell models of HIV-1 latency and in primary CD4^+^ T cells from PLWHIV (Rao et al., 2021), possibly via the activation of NF-κB signalling upon DDX3 depletion. The depletion of DDX3 also resulted in a partial block of nucleocytoplasmic export of the US vRNA, the activation of innate immune signalling pathways, IFN-β production and downregulation of the host proteins BIRC5 (Survivin) which in combination resulted in the selective cell death of HIV-1 US vRNA-expressing cells and a decrease in the size of the inducible viral reservoir (Rao et al., 2021). DDX3’s role in modulating innate immune pathways is also highlighted by studies in dendritic cells where it is shown that DDX3 senses abortive HIV-1 vRNA transcripts to induce type-I interferon immune responses via mitochondrial antiviral signalling protein (MAVS) (Gringhuis et al., 2017; Stunnenberg et al., 2020).

#### 3.1.2 DDX1

Also in 2004, DDX1 was identified as another DEAD-box protein that was a co-factor of Rev (Fang et al., 2004). First characterised as a gene that is overexpressed in retinoblastoma and neuroblastoma (Godbout et al., 1998), DDX1 is involved in processes of pre-mRNA processing such as 3′-end cleavage and polyadenylation (Bleoo et al., 2001). Unique amongst the DEAD-box family proteins, it contains a SPRY domain that could influence its functionality. DDX1 also interacts with the RelA (p65) subunit of NF-κB and is recruited to NF-κB-binding promoter sequences (Ishaq et al., 2009), sequences which are also present in the HIV-1 LTR (Cary et al., 2016). DDX1 was found to be a cellular co-factor of HIV-1 Rev using yeast and mammalian two-hybrid screens (Fang et al., 2004). In mammalian Cos-1 and HEK293 cells, siRNA-mediated depletion of DDX1 resulted in altered subcellular fractionation of Rev by increasing its abundance in the cytoplasm and decreased viral production. Conversely, overexpression of DDX1 resulted in increased viral gene expression. DDX1 levels in astrocytes also were found to contribute to aberrant splicing of HIV-1 vRNAs (Fang et al., 2005). A mutation in the viral Tat protein disrupts Rev-DDX1 binding, indicating that Tat plays an important role in Rev function (Lin et al., 2014). DDX1 promotes the oligomerisation of Rev on the RRE (Robertson-Anderson et al., 2011), and this is mediated by RNA chaperone activity of DDX1 whereby the binding of DDX1 to the HIV-1 US vRNA induces a structural change in the conformation of the RRE that facilitates Rev binding to the RRE.

#### 3.1.3 DDX5

DDX5, also known as p68, is an RNA helicase with characterised roles in splicing, microRNA processing and transcription regulation (Liu, 2002; Wilson et al., 2004; Dardenne et al., 2014). DDX5 is very closely related to DDX17 (a.k.a. p72) and the proteins have 90% homology in their core regions (Lamm et al., 1996). Although primarily localised in the nucleus, DDX5 also has nucleocytoplasmic shuttling ability (Wang et al., 2009). With the discovery of the roles of DDX1 and DDX3 in the nucleocytoplasmic export of the HIV-1 US vRNA and the presence of a nuclear export signal on DDX5, it was speculated that DDX5 would also play a role in vRNA export (Zhou et al., 2013). Indeed, DDX5 was found to co-localise with Rev (Yasuda-Inoue et al., 2013b) and bind to Rev in an RNA-dependent manner to promote the export of RRE-containing transcripts (Zhou et al., 2013). The effect of siRNA-mediated depletion of DDX5 on viral release has been disputed in the past. In one study, siRNA-mediated depletion of DDX5 resulted in increased viral release, a phenotype hypothesised to be due to the increased DDX17 expression upon DDX5 knockdown (Naji et al., 2012). In this study, a combined DDX5 and DDX17 knockdown resulted in decreased viral release (Naji et al., 2012). However, three other reports demonstrate that a knockdown of DDX5 reduce viral production (Zhou et al., 2013; Williams et al., 2015; Sithole et al., 2020). In an siRNA-mediated screen to identify RNA helicases involved in HIV-1 replication, a depletion of DDX5 was found to result in decreased viral production and infectivity (Williams et al., 2015). Follow up work from the same group demonstrated that DDX5 binds to HIV-1 Tat giving it a competitive advantage for binding to the positive Transcription Elongation factor-b (P-TEFb), which is critical for HIV-1 transcription elongation, over the host protein HEXIM1 that sequesters P-TEFb (Sithole et al., 2020). This positive role of DDX5 on HIV-1 transcription was found to be independent of DDX17 and dependent on the DEAD-box domain of DDX5 (Sithole et al., 2020). In macrophages transduced with HIV-1 Vpr, increased levels of DDX5 were observed by mass spectrometry (Barrero et al., 2013).

#### 3.1.4 DDX17

Although DDX5 and DDX17 are very closely related and can even exist as monomers, homodimers or heterodimers (Ogilvie et al., 2003), in the context of HIV-1 replication, they play very distinct roles, even though both proteins are involved in alternative splicing. The first report on the role of DDX17 in HIV-1 replication came from the finding that DDX17 acts as a co-factor of the host zinc-finger antiviral protein (ZAP) that degrades MS vRNA (Zhu et al., 2011). Using a comparative proteomics approach, DDX17 was found to interact with Rev, implying that it is also involved in the nucleocytoplasmic export of HIV-1 US vRNA (Naji et al., 2012). In the same study, an siRNA-mediated knockdown of Rev resulted in decreased viral production and lower SS vRNA and US vRNA levels, with the effect on US vRNA being more pronounced (Naji et al., 2012). The finding that siRNA-mediated knockdown of DDX17 reduces viral production was also observed by two other groups (Williams et al., 2015; Sithole et al., 2018). shRNA-mediated knockdown of DDX17 altered the ratios of unspliced to spliced HIV-1 vRNAs (Lorgeoux et al., 2013). This role of DDX17 on HIV-1 splicing was later found to be mediated by the interaction of DDX17 with splicing factors that are critical for the selection of the A4/5 splice site cluster on the HIV-1 US vRNA (Sithole et al., 2018). This function of DDX17 on HIV-1 splicing was found to be independent of DDX5 and critical for HIV-1 replication (Sithole et al., 2018). In a proteomic screen using bioID, DDX17 was also found to interact with Gag, an association also validated by co-immunoprecipitation (Le Sage et al., 2015). This is in line with the finding that DDX17 is involved in Gag processing by modulating Gag-Pol frameshifting (Lorgeoux et al., 2013). DDX17 is also demonstrated to be involved in HIV-1 vRNA packaging with overexpression of DDX17 resulting in increased genomic RNA packaging (Lorgeoux et al., 2013).

#### 3.1.5 DDX2

Following the export of the vRNAs into the cytoplasm, The DEAD-box proteins also influence cytoplasmic stages of gene expression such as viral protein translation. DDX2, a. k.a. eukaryotic initiation factor 4A (eIF4A), is a helicase that has very well-characterised functions in the initiation of eukaryotic translation as a part of the heterotrimeric protein complex eIF4F. It has two isoforms, eIF4A1 (DDX2A) and eIF4A2 (DDX2B) (Rogers et al., 2002). During cap-dependent translation initiation, eIF4A, as part of a multi-protein complex with initiation factors eIF4G, eIF4E, eIF4B and eIF4H, unwinds secondary structures in mRNA, thereby facilitating the binding of the ribosomal subunits to the mRNA (Rogers et al., 2002). The genomic HIV-1 US vRNA has two translation start sites that generate two isoforms of Gag: AUG1 generates the p55 isoform via a cap-dependent mechanism and AUG2 generates the p40 isoform via an IRES-mediated, cap-independent mechanism (de Breyne et al., 2012; Monette et al., 2013; Amorim et al., 2014). Using a trans-dominant negative mutant of eIF2A that strongly inhibits ribosomal scanning, it was demonstrated that eIF4A was required for translation initiation from both of these start codons, indicating a role for eIF4A in translation initiation of the HIV-1 US vRNA for both cap-dependent and IRES-mediated mechanisms (de Breyne et al., 2012). Interestingly, the removal of the 5′UTR reduced the dependency of Cap-dependent translation on eIF4A (de Breyne et al., 2012). In a tandem affinity purification and mass spectrometry analysis, eIF4A1 was identified as a host protein present with the HIV-1 proteins Gag, Gag/Pol, Env, and Nef as a part of an HIV-1 RNP complex also containing host protein Staufen1 (Milev et al., 2012). shRNA-mediated downregulation of eIF4A2 in a T cell-derived MT4C5 cell line resulted in reduced viral cDNA synthesis and a decrease in the production of replication-competent virus (Ndzinu et al., 2018).

#### 3.1.6 DDX6

DDX6, a. k.a. Rck/p54, is a protein present in P-bodies and stress granules that have characterised functions in microRNA-induced gene silencing, mRNA decay and the suppression of translation (reviewed in (Presnyak and Coller, 2013)). DDX6 is implicated to be an oncogene in colorectal cancer and lymphoma (Nakagawa et al., 1999; Lin et al., 2008) and has characterised roles in the replication of Flavivurises and innate immune responses to infections (Miyaji et al., 2003; Scheller et al., 2009; Göertz et al., 2019; Zhang R. et al., 2021). In the context of HIV-1 infection, two separate reports have demonstrated that depletion of DDX6 results in increased viral production and translation of HIV-1 vRNA (Chable-Bessia et al., 2009; Nathans et al., 2009). This inhibitory effect of DDX6 on HIV-1 replication alludes to its roles in the microRNA pathway, and specifically, its interaction with miR-29a that suppresses HIV-1 translation by enhancing the association of US vRNA with P bodies. A disruption of the P-bodies by DDX6 depletion released the US vRNA, promoted its translation and enhanced the production of viral proteins (Nathans et al., 2009). Interestingly, siRNA-mediated DDX6 depletion also resulted in latency reversal as measured by p24 production in primary cells from three PLWHIV donors under suppressive cART, indicating a post-transcriptional control of HIV-1 latency (Chable-Bessia et al., 2009). DDX6 also plays a role in capsid assembly independent of vRNA packaging and DDX6-depletion disrupted Gag multimerization at the plasma membrane. DDX6 depletion also reduced the production of infectious HIV-1 from *in vitro* infected CD4^+^ T cells (Reed et al., 2012). DDX6 was also identified as a protein that interacts with the HIV-1 gp120 protein via affinity tagging/mass spectrometry studies (Jäger et al., 2012). Interestingly, studies characterising the locations of HIV-1 integrations in cART suppressed PLWH longitudinally, demonstrated clonal expansion of cells with HIV-1 DNA integrated in the DDX6 gene, suggesting an as of yet uncharacterised role for DDX6 in proviral integration or clonal expansion of infected cells (Maldarelli et al., 2014).

### 3.2 DEAD-Box Helicases Belonging to the HIV-1 Protein Interactome

Multiple studies using proteomic analysis to identify host factors involved in HIV-1 replication have identified DEAD-box proteins as interactors of HIV-1 viral proteins. The hits from these studies that were not earlier discussed in this review and their putative roles in HIV-1 viral replication are discussed in the following sub-section:

#### 3.2.1 DDX18

The function of DDX18 in humans is not very well characterised. However, it is known to be activated by Myc (Grandori et al., 1996), is reported to aggravate gastric cancer by modulation of miRNA-21 biogenesis (Zhang Y. et al., 2021) and is associated with poor outcomes in breast cancer patient tumours (Redmond et al., 2015). DDX18 is upregulated in ACH2 cells (T-cell-derived cell lines containing an integrated copy of the HIV-1 provirus) upon latency reversal as well during viral latency in these cells (Krishnan and Zeichner, 2004). DDX18 is also upregulated in a neuroepithelial-like stem (NES) cell line upon treatment with HIV-1 Tat. Proteomic analysis using GST-tagged HIV-1 Tat also identified DDX18 as a Tat-interacting protein, implying that DDX18 might play a role in Tat-mediated transcription or post-transcriptional events following transcription initiation (Gautier et al., 2009). DDX18 was also identified as a Gag-associated cellular factor by proteomic analysis (Roy et al., 2006).

#### 3.2.2 DDX19

DDX19, is a DEAD-box helicase-containing protein that has well-characterised functions in mRNA nucleocytoplasmic export (Folkmann et al., 2011), translation termination and stabilising ribosome complexes with translation elongation factors (Gross et al., 2007; Mikhailova et al., 2017). In humans, two genes code for DDX19 proteins, DDX19A and DDX19B that have 95% homology. In the context of viral infection, DDX19 is also involved in the modulation of innate immune responses (Li et al., 2015; Zhang et al., 2019). Although a defined function for DDX19 in HIV-1 infection has not yet been reported, DDX19A was co-purified with the HIV-1 pre-integration complex, indicating that it may have an as of yet uncharacterised function in the early stages of HIV-1 viral replication (Raghavendra et al., 2010). In studies characterising the Tat-interactome, DDX19 was also found to be a Tat-interacting protein (Gautier et al., 2009). Further work is required to map out the putative molecular mechanisms by which DDX19 may play a role in HIV-1 replication.

#### 3.2.3 DDX21

DDX21, also known as RNA helicase II (RHII/Gu), is an RNA helicase primarily localised in the nucleolus and plays a critical role in ribosomal RNA (rRNA) processing (Flores-Rozas and Hurwitz, 1993; Henning et al., 2003). It also associates with various species of RNA involved in the formation of RNPs, including small nucleolar RNAs (snoRNAs) and 7SK RNA and, via an interaction with the latter, facilitates the release of P-TEFb to promote RNA Pol II-mediated transcription (Calo et al., 2015; Sloan et al., 2015). Interestingly, DDX21 was also a hit in a study characterising the interactome of HIV-1 Tat, which also mediates the release of P-TEFb via interaction with 7SK RNA (Gautier et al., 2009). In ACH2 cells reactivated with PMA, DDX21 was upregulated within 30 min post-induction (Krishnan and Zeichner, 2004). DDX21 is also involved in Rev function. Co-immunoprecipitation analysis first demonstrated that DDX21 associates with the HIV-1 protein Rev (Naji et al., 2012). DDX21 was later found to colocalise with Rev in the nucleolus and the perinucleolar region (Yasuda-Inoue et al., 2013b) and that the DDX21 stimulates Rev-RRE binding (Hammond et al., 2018). In a tandem affinity purification and mass spectrometry analysis, DDX21 was identified as a component of the HIV-1 RNP complex containing host protein Staufen1 and HIV-1 proteins Gag, Gag/Pol, Env and Nef (Milev et al., 2012). siRNA-mediated knockdown of DDX21 in TZM-bl cells infected with HIV-1 resulted in a 75% decrease in p24 release and an increase in intracellular US and MS vRNAs levels, but with minimal effects on the expression of intracellular Gag and Env proteins (Naji et al., 2012). These findings implicate a function for DDX21 in multiple steps of the HIV-1 replication cycle.

#### 3.2.4 DDX46

DDX46, a. k.a., Prp5, plays many roles in pre-mRNA splicing, both before and during pre-spliceosome assembly (Kosowski et al., 2009). Apart from DDX46’s roles in mRNA splicing, a recent study reported that DDX46 is also involved in the host innate immune response. Specifically, DDX46 negatively regulated innate antiviral signalling by recruiting the m^6^A “eraser” ALKBH5 to induce the nuclear retention of transcripts of MAVS, TRAF3, and TRAF6 that are involved in the innate antiviral response (Dang et al., 2019). DDX46 was found to interact with HIV-1 Tat, implicating a role for DDX46 in the transcriptional activation or the co-transcriptional processing of the HIV-1 vRNA (Gautier et al., 2009). Given the emerging roles of the m^6^A epitranscriptomic modification on HIV-1 RNA metabolism (reviewed in (Riquelme-Barrios et al., 2018)) as well as the evidence that DDX46 interacts with Tat, it would be interesting to investigate the possible roles of DDX46 on HIV-1 replication.

#### 3.2.5 DDX39A and DDX39B

DDX39A (a.k.a. URH49 or DDX39) and DDX39B (a.k.a. UAP56) are paralogs of each other and are involved in pre-mRNA splicing and spliceosome assembly (Shen et al., 2007). They also mediate mRNA export, with DDX39A a component of the AREX (alternative mRNA export) complex and DDX39B a part of the TREX (TRanscription-EXport) complex (Yamazaki et al., 2010). In two studies using affinity purification followed by mass spectrometry to identify factors associated with the HIV-1 US vRNA, DDX39B was found to be associated with the US vRNA (Kula et al., 2011; Milev et al., 2012). The HIV-1 protein Rev is reported to inhibit the association of DDX39B with RRE-containing spliced transcripts, preventing the recruitment of the TREX complex to these RNAs (Taniguchi et al., 2014). Further work is necessary to understand the role of DDX39B in HIV-1 vRNA trafficking. DDX39A is a negative regulator of innate immune signalling by binding to antiviral transcripts and sequestering them in the nucleus to limit their expression (Shi et al., 2020). Interestingly, DDX39A was also induced upon HIV-1 infection in primary CD4^+^ T cells in a study that characterised the nuclear proteome of viral-infected cells (DeBoer et al., 2014). Unpublished work by Borsch et al. identifies DDX39A as a hit in an siRNA-mediated screen to identify host factors that can inhibit HIV-1 replication. DDX39A was also found to be upregulated in ACH2 cells during viral latency, implicating a role for it in viral persistence (Krishnan and Zeichner, 2004).

#### 3.2.6 DDX56

DDX56 is primarily localised to the nucleolus and is involved in ribosome biogenesis and nucleocytoplasmic export of RNAs (Zirwes et al., 2000). DDX56 plays a role in the innate antiviral immune response and is recruited by viruses such as Went Nile Virus, and infection with WNV results in the relocalisation of DDX56 from the nucleolus to viral assembly sites (Reid and Hobman, 2017; Li et al., 2019). HIV-1 Rev was found to interact with DDX56 using immunoprecipitation analysis (Yasuda-Inoue et al., 2013b). DDX56 also co-localised with Rev and resulted in an alteration of Rev’s subcellular localisation from the nucleolus to the nucleus (Yasuda-Inoue et al., 2013b). Whether these effects of DDX56 regulate Rev function and whether they are direct or indirect are yet to be elucidated.

#### 3.2.7 DDX47

DDX47 is localised to the nucleolus and is involved in ribosome biogenesis, specifically, in rRNA processing (Sekiguchi et al., 2006). DDX47 was identified as a protein that co-immunoprecipitated with HIV-1 Rev (Naji et al., 2012), indicating that DDX47 may be involved in HIV-1 US and SS vRNA export. siRNA-mediated silencing of DDX47 resulted in a modest reduction of released virus as measured by p24 levels in the supernatant, but no reduction in the intracellular levels of Gag and Env expression (Naji et al., 2012). Whether this effect of DDX47 on HIV-1 replication is direct or indirect remains to be characterised.

#### 3.2.8 DDX24

DEAD-box proteins like DDX24 can also influence steps in the HIV-1 life cycle downstream of translation such as vRNA packaging. Although DDX24 contains the motifs that are conserved in all DEAD-box proteins and is recognised as a putative RNA helicase, little homology can be found between this protein and other DEAD-box proteins (Zhao et al., 2000). DDX24 is an interferon-stimulated gene that behaves as a negative regulator of RIG-I-like receptor (RLR)-mediated signalling and can suppress viral RNA-dependent interferon production (Ma et al., 2013). Proteomic analysis to identify HIV-1 Gag-associated cellular factors identified DDX24 as an interactor of Gag (Roy et al., 2006). Follow-up studies from the same group demonstrated that although the siRNA-mediated knockdown of DDX24 did not reduce the amount of p24 released from infected cells, DDX24 depletion significantly reduced viral infectivity of the released virus due to impaired vRNA packaging (Ma et al., 2008). DDX24’s functions in vRNA packaging were only observed in the context of vRNA exported by Rev-RRE-mediated nucleocytoplasmic export (Ma et al., 2008). This effect is likely mediated by an interaction of DDX24 with Rev (Ma et al., 2008), an association also identified in another study (Naji et al., 2012). A knockdown of DDX24 resulted in a modest increase in nucleocytoplasmic export of the vRNA, but the more striking effects are observed on vRNA packaging (Ma et al., 2008).

#### 3.2.9 DDX20

The products of vRNA translation can also influence the expression of DEAD-box proteins, for example, the Vpr-mediated downregulation of DDX20 (Greenwood et al., 2019). DDX20 is involved in small nuclear ribonucleoprotein (snRNP) biogenesis, critical for spliceosome formation, a function mediated by an interaction with the survival motor neuron (SMN) complex (Campbell et al., 2000). By using an affinity tagging and mass spectrometry approach, DDX20 was identified as an interactor of the HIV-1 accessory protein Vpr (Jäger et al., 2012). A later study demonstrated that DDX20 is directly downregulated by Vpr by DCAF1/DDB1/CUL4 E3 ubiquitin ligase-mediated degradation (Greenwood et al., 2019) during viral replication. Microarray-based studies to identify mRNA and miRNA targets involved in viral latency identified DDX20 as being significantly upregulated in conditions of viral latency in comparison to during active viral replication (Yang et al., 2015), a finding probably observed due to the Vpr-mediated depletion of DDX20 during active viral replication. Given that DDX20 is targeted for depletion by Vpr, it is interesting to speculate that DDX20 plays an inhibitory role during viral replication. Further work in this area is necessary to characterise the potential of targeting DDX20 to inhibit HIV-1 replication.

#### 3.2.10 DDX27

DDX27 is primarily localised in the nucleolus and is reported to play a role in ribosome biogenesis by interacting with the PeBoW complex that is responsible for the generation of 5.8 and 28s rRNAs (Kellner et al., 2015). It is also associated with poor prognosis in gastric, breast and colorectal cancers (Tsukamoto et al., 2015; Tang et al., 2018; Li et al., 2021). DDX27 was present in an HIV-1 RNP that contained the host protein Staufen1 and HIV-1 proteins Gag, Gag/Pol, Env and Nef (Milev et al., 2012), but no other reports of DDX27 in the context of HIV-1 have as of yet been reported.

### 3.3 DEAD-Box Proteins and the Immune Response to HIV-1

DEAD-box proteins play central roles in host immune responses which could potentially influence HIV-1 gene expression. In this sub-section, we discuss the potential and characterised roles of DEAD-box proteins in the immune response to HIV-1.

#### 3.3.1 DDX58

DDX58 codes for Retinoic acid (RA)-inducible gene I (RIG-I), a core component of the innate antiviral signalling pathway involved in the recognition of cytoplasmic viral nucleic acids. It contains CARD-domain and recognises dsRNA to activate a downstream signalling cascade leading to the production of type I interferons and proinflammatory cytokines (reviewed in (Yoneyama and Fujita, 2009)). The secondary structures in the HIV-1 vRNA are also recognised by RIG-I (Solis et al., 2011; Berg et al., 2012), resulting in restricted viral replication in macrophages (Wang et al., 2013; Wang et al., 2015). The expression of DDX58 is upregulated by Vpr expression in myeloid-derived cells (Zahoor et al., 2015), resulting in the stimulation of >20 ISGs in macrophages and dendritic cells (Nasr et al., 2017). To ensure its own replication, HIV-1 employs mechanisms to evade the recognition of its vRNA by RIG-I. People with chronic HIV-1 infection have lower levels of RIG-I expression in PBMCs than in uninfected individuals (Britto et al., 2013). In macrophages, RIG-I is downregulated by the HIV-1 Protease that targets RIG-I for lysosome-mediated degradation (Solis et al., 2011). In CD4^+^ T cells, although the innate antiviral response is very poorly stimulated by HIV-1 (Doehle et al., 2009), latent CD4^+^ T cells present altered stress kinase signalling, making them more susceptible to cell death (Fong et al., 2017). This susceptibility to cell death has been exploited in one study using RIG-I agonists to selectively target HIV-1 infected cells (Li et al., 2016).

#### 3.3.2 DDX41

DDX41 is also involved in innate immune responses and has been identified as an adaptor for STING involved in the sensing of cytosolic DNA (Zhang et al., 2011). DDX41 is involved in pre-mRNA splicing and has tumour suppressor activity, with mutations in DDX41 being associated with Myelodysplastic syndromes (MDS) (Polprasert et al., 2015). During retroviral replication, a DNA/RNA hybrid is generated as the first step of reverse transcription. One report showed that DDX41 senses this DNA/RNA hybrid of mouse leukaemia virus (MLV) to trigger innate immune responses and the induction of IFN-β (Stavrou et al., 2018). They also demonstrated that in bone marrow-derived macrophages and dendritic cells from DDX41 knock-out mice, lower levels of IFN-β were produced than in the control (Stavrou et al., 2018). In a small compound library screen to identify HIV-1 suppressing compounds, the drug Filgotnib was identified as a drug that inhibited HIV-1 replication by inhibiting HIV-1 RNA splicing, but also inducing the intron retention of multiple genes including DDX41 (Yeh et al., 2020). This finding implies that the therapeutic targeting of DDX41 might induce aberrant HIV-1 splicing to inhibit replication.

#### 3.3.3 DDX42

DDX42 is an RNA chaperone that, apart from RNA-helicase activity common to all DEAD-box proteins, also has protein displacement and RNA annealing activities (Uhlmann-Schiffler et al., 2006). Although DDX42 has been linked to mRNA splicing (Will et al., 2002), the cellular functions of DDX42 remain largely uncharacterised. G-quadruplexes (G4) are secondary structures in nucleic acids that assemble in G-rich sequences that regulate transcription and translation and DDX42 was recently identified as G4-binding protein (Zyner et al., 2019). Recent work demonstrates a role for DDX42 in antiviral innate immunity (Bonaventure et al., 2021). Using a CRISPR knockout library, DDX42 was identified as a host protein capable of inhibiting HIV-1 replication (Bonaventure et al., 2021). A knockdown of DDX42 resulted in increased intracellular HIV-1 DNA accumulation in infected cells and increased susceptibility to infection in primary MDMs and CD4^+^ T cells. Conversely, overexpression of DDX42 inhibited HIV-1 infection. The same study demonstrated that DDX42 also restricts viruses from other families like Flaviviruses (Dengue virus, Zika virus, Yellow fever virus and Chikungunya virus) and coronaviruses (SARS-CoV2). Interestingly, DDX42 depletion did not affect Influenza A virus (IAV), vesicular stomatitis virus (VSV) or measles virus (MeV), implying that the antiviral effect is restricted to certain families of viruses. This new data warrants further investigation to understand the role of DDX42 in antiviral responses, also during HIV-1 infection.

#### 3.3.4 DDX50

DDX50 has high genomic similarity to DDX21 and the two genes are oriented in tandem repeats (Valdez et al., 2002). DDX50 behaves as a viral restriction factor that enhances IRF3 activation and IFN-β production (Han et al., 2017; Pallett et al., 2022). In the context of HIV-1 replication, DDX50 however seems to have a proviral role, since an siRNA knockdown of DDX50 resulted in impaired HIV-1 viral replication in a genome-wide RNAi screen (Zhou et al., 2008). The interplay between innate immune activation and helicase function for DDX50 during HIV-1 replication remains to be characterised.

#### 3.3.5 DDX48

DDX48, a. k.a., eIF4A3, is a component of the exon-junction complex (EJC) and is involved in nonsense-mediated mRNA decay (NMD) (Chan et al., 2004). Using affinity purification combined with mass spectrometry, DDX48 was identified to be contained in the HIV-1 vRNA RNP with the HIV-1 proteins Gag, Gag/Pol, Env and Nef, host protein Staufen1 and another protein integral to NMD, UPF1 (Milev et al., 2012). UPF1 has been shown to influence HIV-1 replication and the post-transcriptional control of viral latency by regulating HIV-1 RNA metabolism, with a depletion of UPF1 reducing viral replication (Ajamian et al., 2008; Rao et al., 2018; Rao et al., 2019). However, siRNA-mediated depletion of DDDX48 demonstrated a negligible effect on HIV-1 gene expression (Pak et al., 2015). What is interesting to note, however, is that DDX48 was identified to be upregulated in elite controllers and long-term non-progressors as compared to chronic progressors in integrative genomic analysis of transcriptional profiles of cells from PLWHIV. This indicates that DDX48 may have an as of yet uncharacterised role in HIV-1 viral control.

#### 3.3.6 DDX25

DDX25, also known as Gonadotropin-regulated Testicular RNA Helicase (GRTH), is expressed specifically in the testes and is critical for spermatogenesis by regulating the mRNA export and translation of target genes essential for sperm maturation (Tang et al., 1999; Sheng et al., 2006). Although no characterised role for DDX25 has been reported for HIV-1, DDX25 was found to be a negative regulator of RIG-I-mediated interferon production and a depletion of DDX25 resulted in reduced intracellular viral loads in cells infected with Dengue virus (Feng et al., 2017). It remains yet to be characterised if DDX25 is also involved in the innate immune response to HIV-1 infection.

#### 3.3.7 DDX43

DDX43, also known as Helicase Antigen Gene (HAGE), is highly expressed in the testes and is also upregulated in multiple tumours of various tissues, with negligible expression in normal tissues (Mathieu et al., 2010). Although a direct for DDX43 in HIV-1 replication has not yet been reported, it was observed that DDX43 was downregulated in CD8^+^ cells from early and chronic HIV patients when compared to samples from uninfected and non-progressive donors (Hyrcza et al., 2007), suggesting that DDX43 may be involved in the cell-mediated immune response to HIV-1.

### 3.4 Other DEAD-Box Proteins That Influence HIV-1 Replication by Unknown Mechanisms

Various DEAD-box-proteins have been reported as hits in siRNA screens to identify host factors that influence HIV-1 replication. In this section, we will briefly discuss these hits that have as of yet uncharacterised mechanisms of action for their effects on HIV-1 viral replication:

#### 3.4.1 DDX10

The cellular function of DDX10 is not very well characterised, although it is implicated to play a role in ribosome biogenesis (Savitsky et al., 1996). It is also abnormally expressed in various cancers and has emerged as a potential target for chemotherapy (Quan et al., 2021). The expression of DDX10 is upregulated upon latency reversal in ACH2 cells (Krishnan and Zeichner, 2004). In an siRNA-mediated screen to identify host proteins required for HIV-1 replication, DDX10 was identified as one of the targets (Brass et al., 2008). Subsequent studies showed that a knockdown of DDX10 decreased both intracellular Gag expression levels as well as viral particle production (Williams et al., 2015). The mechanism of action of the effects of DDX10 on HIV-1 replication is not yet known, but given the evidence that DDX10 influences viral replication, further work in this area is warranted.

#### 3.4.2 DDX23

DDX23, a. k.a., prp28, is a component of the U5 snRNP complex that has a function in pre-mRNA splicing by facilitating conformational changes of the spliceosome (Teigelkamp et al., 1997; Möhlmann et al., 2014). There is evidence that DDX23 is upregulated upon HIV-1 latency reversal in ACH2 cells (Krishnan and Zeichner, 2004) and that it is present in an HIV-1 RNP that contains host protein Staufen1 and HIV-1 proteins Gag, Gag/Pol, Env and Nef (Milev et al., 2012). In an RNAi screen to identify genes required for HIV-1 infection, DDX23 was found to be required for the early stages of HIV-1 replication (König et al., 2008). The precise molecular mechanisms underlying these functions of DDX23 on HIV-1 replications remain yet to be characterised.

#### 3.4.3 DDX28

The host cell function of DDX28 is not very well characterised. It is localised to the mitochondria and the nucleus (Valgardsdottir et al., 2001) and is present in a mitochondrial RNA granule that drives the biogenesis of mitochondrial ribosomes (Antonicka and Shoubridge, 2015; Tu and Barrientos, 2015). In an siRNA-mediated screen to identify RNA helicases required for HIV-1 infection, DDX28 was reported as an essential HIV-1 cofactor (Williams et al., 2015). A knockdown of DDX28 resulted in decreased levels of p24 released in the supernatant as well as reduced infectivity of the released HIV-1 (Williams et al., 2015). The mechanisms underlying DDX28’s effect on HIV-1 replication remain uncharacterised but are interesting to investigate as they might highlight a new link between mitochondrial ribosome biogenesis and HIV-1 replication.

#### 3.4.4 DDX49

The cellular functions of DDX49 were uncharacterised until recently when it was demonstrated to be localised to the nucleolus, regulate the steady-state levels of pre-ribosomal RNA and promote the export of polyA + RNA from the nucleus in a splicing-independent manner (Awasthi et al., 2018). In a genome-wide siRNA screen to identify host factors required for HIV-1 replication, DDX49 was found to be essential for viral production (Zhou et al., 2008). DDX49 was also found to interact with HIV-1 Gag protein using the use of affinity tagging and purification mass spectrometry (Jäger et al., 2012). In microarray analysis, DDX49 was found to be upregulated in donors with viral progression as compared to matched long-term non-progressors (Salgado et al., 2011), suggesting that DDX49 may play a role in HIV-1 disease progression. Further work is required to understand the mechanism of DDX49’s roles in HIV-1 viral production.

#### 3.4.5 DDX52

The host cell functions of DDX52 are not very well understood, but it is speculated to be involved in rRNA biogenesis and in involved in the regulation of c-Myc, therefore being associated with lung cancer, prostate cancer and melanoma (Venema et al., 1997; Wang et al., 2021). The expression of DDX52 was upregulated with 30 min of HIV-1 latency reversal in ACH2 cells, suggesting that it may have a proviral role in HIV-1 replication (Krishnan and Zeichner, 2004). Indeed, in an siRNA screen to identify helicases involved in HIV-1 replication, a knockdown of DDX52 resulted in a decreased viral release as measured by p24 levels in the supernatant on transfected HeLa cells (Williams et al., 2015). Although viral production was reduced, DDX52 depletion did not affect the infectivity of the released virus (Williams et al., 2015), implying that the effect of DDX52 on HIV-1 replication may be before packaging and assembly. Further work is necessary to understand the mechanisms of DDX52’s role in HIV-1 replication.

#### 3.4.6 DDX53

DDX53, a. k.a. cancer-associated antigen gene (CAGE), is highly expressed in tumours and is associated with resistance to anti-cancer drugs (Kim et al., 2010). It also induces cytolytic T lymphocyte activity and promotes cell motility (Shim E. et al., 2006; Shim H. et al., 2006). In a large-scale siRNA mediated screen to host factors with a role in HIV-1 function, a knockdown of DDX53 resulted in a reduction of the amount of intracellular p24 produced as measured by immunofluorescence staining for intracellular p24 (Brass et al., 2008). A further function for DDX53 in HIV-1 replication remains to be studied.

#### 3.4.7 DDX55

DDX55 is localised to the nucleolus and is involved in the assembly of the large ribosomal subunit by binding to the 28s rRNA (Choudhury et al., 2021). In the same siRNA screen that implicated DDX53 as a host factor involved in HIV-1 replication, a knockdown of DDX55 also resulted in reduced intracellular p24 levels (Brass et al., 2008). This suggests that DDX55 is also involved in the HIV-1 life cycle in a yet uncharacterised manner.

#### 3.4.8 DDX60 and DDX60L

DDX60 and DDX60L (DDX60-like) are interferon-inducible genes that promote RIG-I-mediated signalling to control viral infections (Miyashita et al., 2011; Oshiumi et al., 2015). They are homologs that share a 70% sequence conservation in their core regions (Miyashita et al., 2011). Gene-expression profiling revealed that HIV-1 Vpr induced the upregulation of DDX60 in dendritic cells (Zahoor et al., 2015). A genome-wide siRNA screen revealed that depletion of DDX60L resulted in impaired HIV-1 replication (Zhou et al., 2008). This data suggest that rather than functioning as an antiviral factor, HIV-1 may recruit DDX60 and DDX60L to promote its replication. Although DDX60 and DDX60L can inhibit viral replication of other RNA viruses like HCV (Schoggins et al., 2011; Grünvogel et al., 2015), whether they are involved in the innate antiviral immune response to HIV-1 remains to be studied.

## 4 Targeting DEAD-Box Proteins to Inhibit HIV-1 Replication

Viruses belonging to the Flaviviridae family (Zika virus, HCV, Dengue Virus, etc.) encode their own RNA helicases for viral replication, the NS3 protein (Mastrangelo et al., 2012). Poliovirus, Rubella virus and coronaviruses also have viral-proteins with RNA helicase function. Since these proteins are virus-encoded factors, their therapeutic inhibition likely has lesser off-target effects. Indeed, multiple reports describe the successful therapeutic inhibition of the NS3 protein of HCV to inhibit viral replication (Borowski et al., 2002; Maga et al., 2005; Manfroni et al., 2009). However, HIV-1 does not code for its own helicase and therefore must rely on host proteins such as the DEAD-box helicases to mediate multiple stages of the viral life cycle (as described in Section 3). Since DEAD-box helicases are also involved in all steps of RNA biology and are crucial to multiple post-transcriptional processes, the development of DEAD-box protein inhibitors as a treatment for HIV-1 raises the issue of selective toxicity. On the other hand, targeting a more conserved host protein instead of a viral protein to inhibit viral replication could also result in a lower chance of the development of drug resistance. The following inhibitors of DEAD-box proteins have been described, and are discussed in their potential to be used against HIV-1 (Table2).

**TABLE 2:** List of DEAD-box helicase-directed therapeutics and their demonstrated effect on HIV-1.

DEAD-Box Protein	Compound	Effect on HIV-1	References for Link to HIV-1
DDX2 inhibitors	Hippuristanol	Inhibition of cap- and IRES-mediated translation	Plank et al. (2014)
	Pateamine A	HIV-1 can inhibit Pateamine A-induced stress granule assembly	Valiente-Echeverría et al. (2014)
	Silvestrol	—	—
	Hypericin	—	—
	Elisabatin A	—	—
	Allolaurinterolas	—	—
DDX3 inhibitors	Ring expanded nucleoside analogues (RENs)	Inhibit HIV-1 replication in CD4^+^ T cells and monocyte-derived macrophages	Yedavalli et al. (2008)
	Rhodanine derivative small-molecule inhibitors	Inhibit HIV-1 replication in PBMCs	Radi et al. (2012)
			Maga et al. (2011)
	NZ51	—	—
	Ketorolac	—	—
	1,3,4-Thiadiazole Inhibitors	Inhibit HIV-1 replication in PBMCs	Brai et al. (2019)
	RK-33	Induces latency reversal and selective cell death of HIV-1 infected cells by activating innate immune responses	Rao et al. (2021)
	RNA binding site inhibitor 16d	Inhibit HIV-1 replication in PBMCs, Induces latency reversal and selective cell death of HIV-1 infected cells by activating innate immune responses	Rao et al. (2021)
			Brai et al. (2016)
DDX58 (RIG-I) agonists	Acitretin	Reverse viral latency and induce apoptosis in HIV-1 infected cells, not reproduced in follow up studies	Li et al. (2016)
			Garcia-Vidal et al. (2017)

### 4.1 DDX2 Inhibitors

Targeting host cell translation is a strategy being explored to improve cancer treatment (reviewed in (Jiang et al., 2021)), resulting in the characterisation of drugs against the DEAD-box protein DDX2 (eIF4A). One of these drugs is Hippuristanol, a steroid that was isolated from a coral that demonstrated anti-proliferative activities *in vitro* for cancer treatment (Higa et al., 1981) and was later characterised as a selective inhibitor of DDX2 by inhibiting its ATPase activity (Bordeleau et al., 2006). Hippuristanol treatment of HeLa cells transfected with constructs of the HIV-1 resulted in the inhibition of both cap-mediated (Toro-Ascuy et al., 2018) and IRES-mediation translation, indicating that the HIV-1 IRES utilises eIF4A for translation initiation (Plank et al., 2014). Pateamine A is another inhibitor of DDX2-dependent translation that was originally isolated from a marine sponge (Northcote et al., 1991). It inhibits cap-dependent translation by enhancing eIF4A activities, thereby inhibiting eIF4A-eIF4G association, resulting in the formation of stress granules and the effects of this drug are irreversible (Bordeleau et al., 2005; Low et al., 2005). Interestingly, HIV-1 can inhibit Pateamine A-induced stress granule assembly via an interaction with the eukaryotic elongation factor eEF2 (Valiente-Echeverría et al., 2014), thereby demonstrating how HIV-1 can override blocks to ensure translation of its transcripts. Pateamine A also binds DDX48 (Low et al., 2007), although with less affinity than it does DDX2, and can inhibit NMD via this interaction with DDX48 by stabilising the interaction between UPF1 and the EJC (Dang et al., 2009). Rocaglamide A (Roc-A) derivatives such as Silvestrol also reversibly inhibit DDX2 function (Bordeleau et al., 2008). Low and high-throughput screens also identified hypericin and elisabatin A and allolaurinterolas respectively as compounds that inhibited DDX2 activity (Cencic et al., 2012; Tillotson et al., 2017). Although it is evident that HIV-1 requires DDX2 for its replication, eIF4A is also involved in central processes of host cell metabolism. Given the pleiotropic implications of inhibiting DDX2 *in vivo*, none of the current inhibitors which are all in pre-clinical development, has yet reached clinical trials. It remains to be explored whether a therapeutic window can be found with these inhibitors that would result only in the selective death of HIV-1 infected cells with minimum toxicity.

### 4.2 DDX3 Inhibitors

Numerous inhibitors of DDX3 have been described with different mechanisms of action and limited toxicity, many of them specifically to identify new classes of inhibitors for HIV-1. The first specific small-molecule inhibitors of DDX3 were identified using structure-based pharmacophore modelling and molecular docking studies and belonged to a class of rhodanine derivatives (Högbom et al., 2007; Maga et al., 2008). Concurrently, ring expanded nucleoside analogues (RENs) were found able to inhibit the ATP-dependent activity of DDX3 and also suppress HIV-1 replication in CD4^+^ T cells and monocyte-derived macrophages (Yedavalli et al., 2008). Further optimised rhodanine derivative small-molecule inhibitors were then synthesized that could inhibit HIV-1 replication in PBMCs *in vitro* assays (Maga et al., 2011; Radi et al., 2012). These second-generation DDX3 inhibitors that were specifically bound to the RNA-binding site had nanomolar anti enzymatic activity specifically against DDX3, low cellular toxicity and anti-HIV-1 activity by inhibiting viral production (Maga et al., 2011). Next-generation compounds that target the ATPase activity of DDX3 were also synthesized such as NZ51 and RK-33, the latter widely characterised as an anti-tumour compound, with activity against multiple tumour types (Bol et al., 2015a; Xie et al., 2015). RK-33 binds to the ATP-binding site of DDX3 and also has been demonstrated to have broad-spectrum antiviral activity, inhibiting the replication of human parainfluenza virus type 3 (hPIV-3), respiratory syncytial virus (RSV), dengue virus (DENV), Zika virus (ZIKV) and West Nile virus (WNV) (Yang et al., 2020). The Ketorolac salt was also reported to inhibit the ATPase activity of DDX3 (Samal et al., 2015). 1,3,4-Thiadiazole Inhibitors also inhibited the ATPase activity of DDX3 and inhibited HIV-1 replication (Brai et al., 2019). Structure-based drug design approaches using molecular docking against the closed conformation of DDX3 bound to RNA were also used to identify additional DDX3 inhibitors that bound to the RNA-binding site of DDX3 (Fazi et al., 2015). Additional compounds were synthesized that could bind to the DDX3 RNA-binding site and inhibit RNA helicase activity of DDX3, of which one compound (called 16d in (Brai et al., 2016)) demonstrated antiviral activities against wild type HIV-1 in PBMCs, as well as other viruses such as HCV, Dengue virus and West Nile virus (Brai et al., 2016). Importantly, this compound also demonstrated antiviral activity against HIV-1 strains carrying clinically relevant drug-resistance-associated mutations of HIV-1 (Brai et al., 2016). All these data together indicate that DDX3 inhibitors have a high potential to be developed into a new class of antiretrovirals that can control HIV-1 infection. In our own work (Rao et al., 2021), we investigated the effects of DDX3 inhibition in HIV-1 infected cells using RK-33 (that binds to the ATP-binding site of DDX3) and the compound 16d (Brai et al., 2016) (that bound the RNA-binding site). Even though the compounds inhibited DDX3 by different mechanisms, we observed similar results on HIV-1 replication and cellular apoptosis. Specifically, DDX3 inhibition resulted in latency reversal and the activation of innate antiviral signalling pathways resulting in the production of IFN-β. DDX3 inhibition also induced the downregulation of the host protein BIRC5. These effects resulted in the induction of apoptosis in the HIV-1 infected cells. Importantly, in cells from people living with HIV-1, prolonged treatment of cells with these DDX3 inhibitors resulted in the selective cell death of the HIV-1-infected cells and a reduction in the size of the inducible viral reservoir (Rao et al., 2021). These results serve as a proof of concept that, apart from having a potential to be developed as next-generation antiretrovirals, DDX3 inhibitors can also be utilised in HIV-1 curative strategies to clear out the viral reservoir.

### 4.3 DDX58 (RIG-I) Agonists

Similar to DDX3 inhibition, another strategy to selectively clear out the viral reservoir is by the induction of innate immune signalling in infected cells that would lead to their selective cell death and apoptosis. RIG-I agonists have previously been investigated for their potential to induce selective cell death of HIV-1 infected cells, such as the FDA-approved compound acitretin that is used to treat psoriasis (Ortiz et al., 2013). One study demonstrated that acitretin could reverse viral latency and induce apoptosis in HIV-1 infected cells (Li et al., 2016). In CD4^+^ T cells from 12 PLWHIV, *in vitro* treatment with acitretin for 7 days resulted in a decrease in the frequency of HIV-1 DNA levels in these cells (Li et al., 2016). However, follow-up studies from another group could not reproduce these effects of acitretin on either the latency reversal or on the selective killing of HIV-1 infected cells using cell lines and primary CD4^+^ T cell models of HIV-1 latency (Garcia-Vidal et al., 2017). However, both of these studies demonstrated that acitretin could induce RIG-I signalling and was an innate immune modulator. The combination of innate immune modulators with latency reversal agents could result in a stronger activation of innate immune signalling and selective infected cell death. Further studies are needed to investigate agonists not only of DDX58 but also other DEAD-box proteins involved in innate immune signalling for the ability to induce cell death of HIV-1 infected cells.

## 5 Conclusion

**FIGURE 2:**
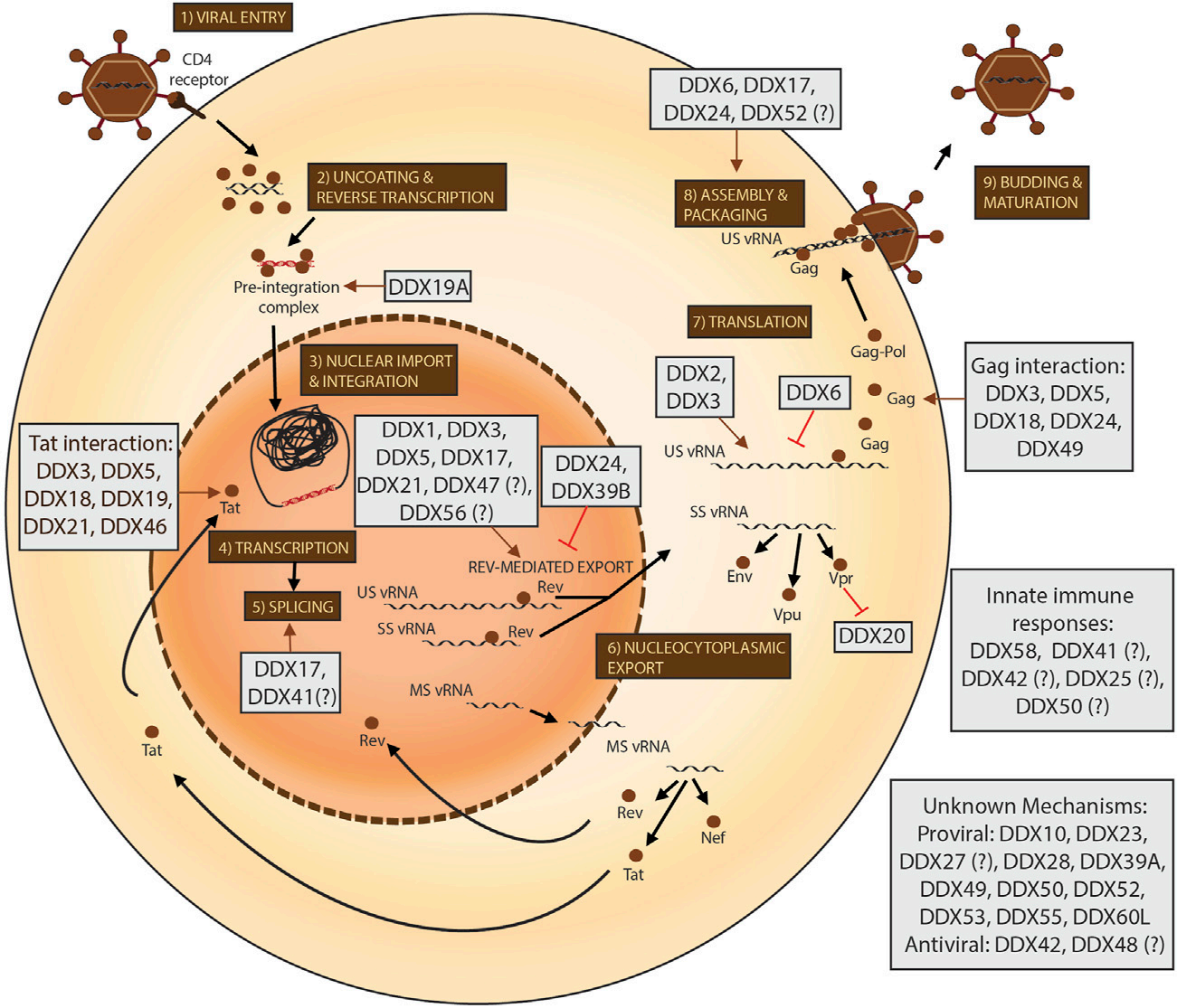
The roles of DEAD-box proteins in the HIV-1 life cycle. The steps of the HIV-1 life cycle are indicated from 1-9. viral RNA is depicted in black and viral DNA in red. Progression of the steps of the life cycle are indicated with black arrows and all viral proteins are depicted as brown circles. The effects of the DEAD-box proteins (in grey boxes) are indicated by either brown arrows for a pro-viral effect and with red lines for an anti-viral effect. DEAD-box proteins with uncharacterised or speculated effects in the life cycle are accompanied by a question mark.

DEAD-box proteins can influence viral replication at almost every stage of the viral life cycle (as depicted in Figure 2). However, a lot of outstanding questions remain, such as whether these proteins have redundant functions in the context of HIV-1 replication, whether they are found in the same RNPs and act as co-factors of each other, whether they bind HIV-1 vRNAs in a sequence-specific manner and what the precise mechanisms of actions of the functions of these proteins on HIV-1 are. Importantly, it also remains to be elucidated whether these proteins play a role in the maintenance of viral latency and could be relevant in the context of the development of curative HIV-1 therapies. The success of structure-based drug design assays for the development of DDX3 inhibitors with high selectivity indices and evidence of anti-HIV-1 activity highlight that targeting DEAD-box proteins to inhibit HIV-1 replication is a viable strategy to control the HIV-1 pandemic.
